# A Comparison of Self-reported Condomless Sex and Yc-DNA Biomarker Data from Young Women Engaged in High Risk Sexual Activity in Kampala, Uganda

**DOI:** 10.1007/s10461-023-04177-y

**Published:** 2023-09-26

**Authors:** Pippa Boering, Janet Seeley, Joshua Buule, Onesmus Kamacooko, Rachel King

**Affiliations:** 1 London School of Hygiene and Tropical Medicine, London, UK; 2 MRC/UVRI and LSHTM Uganda Research Unit, Entebbe, Uganda; 3 Uganda Virus Research Institute, Entebbe, Uganda; 4 University of California, San Francisco, CA USA; 5 INSERM, 60 Rue de Navacelles, 34090, Montpellier, France

**Keywords:** Condomless Sex, HIV Prevention, Sex work, Social desirability bias, Biomarkers

## Abstract

Reporting of condom-use can limit researchers’ understanding of high-risk sexual behaviours. We compared self-reported condom-use with the Yc-DNA biomarker data and investigated potential factors influencing participation in, and reporting of, sexual behaviours. Self-reported data were collected using Audio Computer Assisted Self Interviews (ACASI) and samples for Yc-DNA biomarker were collected using self-administered and health worker-collected vaginal swabs from 644 women (aged 15–24 years) who were not living with HIV. Yc-DNA results and interview data were compared using McNemar-Bowker Analysis and Cohen’s Kappa. Test statistics for Yc-DNA biomarker were calculated. Log Binomial models for Yc-DNA and self-reported results were conducted to assess for association. We found strong evidence (p < 0.001) for a difference between Yc-DNA and self-reported results. 13.7% of participants reported consistent condom-use with all partners, regardless of HIV status. Self-reported condom-use was discordant in 50.0% (n = 206) of cases, when compared to Yc-DNA results. Positive Yc-DNA results were found to be associated with older age (RR 1.36; 95%CI 1.04, 1.76 p = 0.023). Self-reported condom-use with partners with unknown HIV status was associated with higher education (RR 0.76; 95%CI 0.58,0.99 p = 0.043). Sensitivity analysis did not determine difference between methods for controlling for missing data. We found significant under-reporting of condomless sex in the self-reported data when compared to Yc-DNA results.

## Background

The accurate and reliable measurement of high-risk sexual behaviour is essential for the synthesis of representative sexual and reproductive health research, and consequent implementation of context-specific interventions [1, 2]. Methodological challenges regarding self-reported sexual behaviour have resulted in interest in dependable biomarkers to replace or support these data. Y-chromosomal deoxyribonucleic acid (Yc-DNA) biomarkers have been developed to detect DNA unique to the male genome from male epithelial cells and sperm found in the vagina following condomless penetrative sex [3, 4].

Previous studies evaluating Yc-DNA biomarkers have shown that it can provide a reliable alternative to self-reported data, however, these studies have been limited by small sample sizes. Across studies, validity of Yc-DNA has been reported to be between 87 and 100% when tested the day after condomless sex and can be detected with sufficient sensitivity for 14–15 days following condomless sex [5]. Ghanem et al. [6] showed Yc-DNA to have a specificity of 92% in a study of 54 women in the United States. Brotman et al. [7] determined the half-life of Yc-DNA to 3.8 days and could be detected for 15 days in a study of 51 women in Baltimore, MD, USA. This was corroborated by Zenilman et al. [3] who demonstrated Yc-DNA could be detected up to 2 weeks following exposure in a study of 21 women. Yc-DNA detection is influenced by the concentration of DNA present on the swab, as well as personal hygiene practices such as douching [7].

The lack of congruency between self-reported condom-use and Yc-DNA results has been reported in a case-control study conducted by Heffron, Parikh [8] which indicated a third of 428 participants were over-reporting their condom use. Furthermore, two studies conducted among female sex workers (FSW), provided insights into the factors influencing self-reported data. One study conducted among 334 adult (mean age 33.4) FSW in Benin used prostate specific antigen (PSA) and Yc-DNA biomarkers to verify self-reported condom-use [9]. The study found that the under-reporting of condomless sex was similar at two varying timepoints, suggesting that recall bias was not the cause of the difference between self-report and biomarker result. A study with 267 female sex workers in Senegal [10] detected Yc-DNA in 26% of swabs from participants reporting consistent condom use.

Young women who sell sex (YWSS) experience complex biological, socio-cultural, economic and political vulnerabilities which impact their health and wellbeing including their ability to negotiate condom-use [11]. Adolescent girls and young women have a significantly high incidence of HIV, and this risk is compounded when they are engaged in selling sex. Estimates from Kenya indicate that FSW have 10 times the odds of HIV infection compared to other women, with between 20 and 45% of FSW living with HIV [12]. Young women who sell sex in Uganda face several barriers when negotiating condom-use, including power differentials relating to their age and gender inequalities as well as vulnerabilities associated with economic instability and working in a criminalised setting [13].

Sexual behaviour presents a unique challenge to research due to its highly intimate, and in some contexts, stigmatized nature. Both high-risk and risk-mediating sexual behaviours are influenced by broad legal, moral, religious and socio-cultural factors which inform socialised sexual scripts and determine what behaviour is deemed acceptable [14]. This psychosocial influence results in the over-reporting of normative behaviour, and under-reporting of behaviours considered to be stigmatized, illegal or deemed “risky” in research [15]. The power imbalance created in the researcher-researched dynamic may result in conscious or subconscious bias [16, 17]. Further challenges arise when participants are asked to recall events over periods of time or are asked questions which do not reflect their context [18].

We compared self-reported condomless sex and Yc-DNA results among YWSS in Kampala, Uganda to investigate the factors impacting self-reported data in sexual behaviour research.

## Methods

### Study Population

This analysis was conducted using data collected at baseline of the “A Cognitive Behavioural and Structural HIV Prevention Intervention for Young Ugandan Women engaging in High-Risk Sexual Behaviour (ZETRA-Study for zero transmission)” [19, 20]. The study consisted of two arms, with the intervention arm receiving health literacy and technology skill building workshops, and the control arm engaging in standard of care prevention counselling.

A sample of 644 women (aged 15–24 years) who were at risk of HIV agreed to participate in the study and were recruited from women attending the Good Health for Women Project Clinic in Kampala, Uganda. The clinic was open from 2008 to 2020. It was established by the Medical Research Council/ Uganda Virus Research Institute and London School of Hygiene & Tropical Medicine (MRC/UVRI & LSHTM) Uganda Research Unit to study the epidemiology of HIV, sexually transmitted infections (STI) and to implement HIV and STI prevention among women at high risk of HIV acquisition, including female sex workers. The study setting and population are described in more detail in Kasujja et al. [20] and Kamacooko et al. [19].

Women were recruited to the Good Health for Women Project clinic by field workers. They conducted mobilization activities with community peer sex worker-leaders to identify sex workers from commercial-sex locations and enrolled these women to receive care from the clinic irrespective of HIV status. All enrolled women attended quarterly follow-up visits including comprehensive HIV prevention and treatment services described above, regardless of participation in the trial.

Inclusion criteria for the ZETRA trial included: HIV-negative women aged 15–24 years, being sexually active (defined as participants who stated that they had sex in the last 3 months) and having engaged in any form of transactional sex at least once in the last 3 months, agreeing to participate in intervention sessions and to all study procedures and interviews planned over 18 months of follow-up.

### Data Collection

Data used in the analysis for this paper were collected at baseline. Participants principally provided data using Android electronic tablets (hand-held computers) using Computer Assisted Self Interviews (ACASI).

Demographic variables were collected at baseline using ACASI. The socio-demographic variables: age of participant, level of education, and religion, were included in the analysis. Furthermore, participants were asked the number of times they engaged in condomless sex over the last month for partners of unknown HIV status and HIV negative partners. This was recorded in grouped data as never, less than 5, 5–10 or more than 10 times.

### Yc-DNA Collection and Analysis

Vaginal Yc-DNA samples were collected as part of study procedures for all participants. Yc-DNA collection was not completed when a participant reported they were menstruating. Both self- and health worker- administered high vaginal swabs were collected using dry Dacron® swabs. The vaginal swabs were stored dry at − 20 °C and transported in a cooler box with ice packs to maintain the temperature. The samples were tested by a team at UVRI-Abbott research laboratory in Entebbe. The Promega DNA iQ extraction kit was used. The swab head was placed in 0.25mL of lysis buffer containing 15µL of 1 M dithiothreitol (DTT) solution and incubated at 70 °C for 30 min. The sample was centrifuged in a 1.5ml tube at 14,000 rpm for 1 min to remove excess liquid. 7µL of resin was added to the supernatant, vortexed for 2 s and then incubated for 5 min with interval mixing every minute, then placed in a magnetic stand to separate the resin-attracted DNA from the lysis solution. This was followed by three washing steps with wash buffer coupled with vortex-mixing between each step. The resin-DNA complex was allowed to air dry for five minutes, then 40µL of elution buffer was added and mixed gently by tapping the bottom of the tube. This was followed by an incubation step at 65 °C for five minutes. The mixture was placed in a magnetic stand while still hot and the elution buffer containing the Yc-DNA was pipetted into a clean 1.5ml tube for storage at -20 °C until it was used for PCR amplification.

To analyse presence of Yc-DNA, an in-house Multiplex PCR assay was used on the stratagem PCR machine (Agilent technologies, USA) to simultaneously detect Testis-Specific Protein, Y-Encoded (TSPY-1 – DYS14) as well as GAPDH, a house keeping gene used as a nucleic acid reference maker. No false positive results were found.

### Data Analysis

Data analysis was conducted using STATA 15.0 (Stata Corp, College Station, TX, USA). Descriptive analysis of all variables was conducted, and subsequently the self-report condom-use data were compared with the Yc-DNA results. Finally, additional analysis was conducted to investigate factors associated with self-reported condomless sex as well as positive Yc-DNA results.

### Descriptive Analysis

Initial analysis consisted of exploration of socio-demographic variables, including distribution of variables in the sample. Normality of distribution was assessed using histograms, and extent of missing data were investigated using tabulation. Distribution of Yc-DNA results was assessed for all demographic and behavioural variables. Patterns for missing data were evaluated. Distribution of variables were tabulated and association with Yc-DNA was assessed using the Chi-squared test.

### Comparison Between Yc-DNA and Self-Reported Condom-Use

Initial descriptive comparison was conducted using 2 × 2 tables to examine the distribution of Yc-DNA results and self-reported condom-use with both unknown HIV status and negative HIV status. Additionally, these variables were combined to determine condom-use with all partners in the past month, and this was compared to Yc-DNA results. McNemar Chi-squared test was used to assess the difference between the two forms of self-reported condom-use. Comparative analysis was conducted using the McNemar-Bowker test to compare Yc-DNA with self-reported condomless sex with partners of negative HIV status, as well as partners with unknown HIV status. The McNemar-Bowker test is an extension of McNemars test and can be used for paired categorical data with more than 2 categories. It is an omnibus statistical test, enabling it to test for significance of several parameters in 1 model [21]. Furthermore, sensitivity, specificity, and positive predictive value were calculated using diagnostic test analysis. Agreement between methods was assessed using Cohen’s Kappa as well as percent agreement.

### Log Binomial Models

Analysis of factors associated with positive Yc-DNA results, as well as confounding, was conducted using log binomial models. Log binomial models were also conducted to assess for factors associated with self-reported condom-use with both partners of negative HIV status as well as unknown HIV status. The variables for Yc-DNA and condom-use were recoded to be binary. Yc-DNA was recoded to be 0 = Negative and 1 = Positive. Both self-reported variables were recoded to 0 = Did use condom and 1 = Did not use condom. All models were adjusted for age, considered to be an a priori confounder.

Additionally, log binomial models were designed to assess factors influencing self-reported condomless sex in the past month, as well as Yc-DNA results, following crude log binomial models for each variable. These models included the a priori confounder age and distal socio-cultural factors of level of education and religion. The three models retained variables that indicated a strong (RR > 1.5) or statistically significant (p < 0.05) effect. Multicollinearity and sparse data were assessed. Wald test results were used to assess variable contribution to the model. Furthermore, statistical interaction between variables was assessed.

### Missing Data

Missing Yc-DNA results and missing results for self-reported condomless sex with partners who are HIV negative were initially addressed by restricting the log binomial models to only complete cases. Subsequently, multiple imputations were conducted assuming data were missing at random (MAR), and the analysis included all variables. Stata’s “mi impute logit” command generated 10 imputations for each regression model. Subsequent estimates of the odds ratios were obtained according to Rubin’s rules [22]. The robustness of conclusions was assessed using sensitivity analysis.

### Ethics Approval and Consent

This analysis was given ethics approval by the Ethics Committee at the London School of Hygiene and Tropical Medicine (LSHTM) (ref: 27,359). Data sharing for the purpose of this analysis was approved by the Medical Research Council/Uganda Virus Research Institute and London School of Hygiene & Tropical Medicine Uganda Research Unit. The intervention trial was approved locally by the Uganda Virus Research Institute Research Ethics Committee and the Uganda National Council for Science and Technology. All participants provided written informed consent forms and data have been anonymised to ensure participants confidentiality. Participants were given transport refunds of Ugx 20,000 (~$5.19 based on 2022 conversion rates) for every visit to the clinic, and medical treatments were offered to participants if clinically indicated.

## Results

The characteristics of participants who were enrolled in this study (n = 635) are shown in Table 1, as well as the distribution of positive Yc-DNA results, missing data and associated Chi-squared p-value for each characteristic. All data were found to be normally distributed. At baseline, the mean age of participants was 19.9 (SD = 2.50), 40.6% of participants had not completed formal education, including primary and secondary education, and the majority (37.9%) were Roman Catholic. Half (47.8%) of the participants’ most recent sexual encounter reported at the time of data collection was with a casual sexual partner, and 53.9% of participants did not know their most recent sexual partner’s HIV status. No patterns of missing data were found.

**Table 1:** Participant Characteristics and Distribution of Positive Yc-DNA results (n = 635)

Variable	Overall N n(col%)	Positive Yc-DNA N(of 491 Yc-DNA results %))	χ² P-value
All Participants	635(100)	194 (39.5)	
Age of the participant			
15–18	202(31.8)	55(11.2)	0.155
19–21	241(38.0)	66(13.4)	
22–24	192(30.2)	73(14.9)	
Level of Education			
No Education	258(40.6)	84(17.1)	0.504
Primary Level	249(39.2)	72(14.7)	
Secondary or Tertiary	128(20.2)	38(7.73)	
Religion			
Born Again	82 (12.9)	27(5.50)	0.670
Catholic	240(37.9)	72(14.7)	
Moslem	182(28.7)	46(9.37)	
Protestant	116(18.3)	41(8.35)	
Other Religion	10 (1.58)	6(1.22)	
No Religion	4(0.63)	2 (0.41)	
Missing	1(0.2)	0(0.0)	
Most Recent Sexual Partner			
Casual	227(47.8)	83(16.9)	0.726
Steady	117(24.6)	49(9.98)	
Spouse	131(27.6)	54(11.0)	
Missing	22(3.5)	8(1.63)	
Most Recent Sexual Partners HIV Status			
HIV Positive	9(1.9)	1(0.20)	0.237
HIV Negative	210(44.2)	91(18.5)	
Don’t Know	256(53.9)	94(19.1)	
Missing	22(3.5)	8(1.62)	
Condomless Sex with Partner who is HIV Negative in Past Month			
Never	53(11.6)	17(3.46)	0.817
Between 1–5	148(32.2)	58(11.8)	
Between 5–10	82(17.9)	32(6.51)	
More than 10	49(10.7)	21(4.28)	
Don’t Know	127(27.7)	48(9.78)	
Missing	41(6.5)	18 (3.67)	
Condomless Sex with Partner with Unknown HIV Status in Past Month			
Never	237(49.9)	96(19.6)	0.849
Between 1–5	124(26.1)	51(10.4)	
Between 5–10	37(7.8)	14(2.85)	
More than 10	28(5.9)	10(2.04)	
Don’t Know	49(10.3)	15(3.05)	
Missing	22(3.5)	8(1.63)	

*Chi-squared Test result for each characteristic and Yc-DNA result

Yc-DNA swabs were collected from 491 participants, and 194 (39.5%) participants had positive results. There were 79 (19.3%) invalid results and 218 (44.4%) participants had negative results. There were 144 participants (22.7%) with missing Yc-DNA results. Self-reported consistent condom-use with all partners in the past month was 13.7%. When compared to Yc-DNA results, 50.0% (n = 206) of self-reported results did not correspond with Yc-DNA results. Self-reported condom-use with partners who were HIV negative was 11.6% (n = 53), however most (32.2%) participants reported having condomless sex 1–5 times in the past month. There was a significant percentage (6.5%) of missing results for self-reported condom-use with partners who were HIV negative. Half (49.9%) of the participants reported never having condomless sex with partners with unknown HIV status, while 26.1% of participants reported having had condomless sex 1–5 times. The Chi-squared test conducted comparing each characteristic with Yc-DNA did not highlight any evidence for an association of Yc-DNA with any characteristic.

### Comparison Between Self-Reported Condom-Use and Yc-DNA

Initially, comparison of self-reported condom-use and Yc-DNA results was conducted using descriptive 2 × 2 tables to show the distribution of Yc-DNA results compared to self-reported condom-use with all partners in the past month (Table 2). Furthermore, self-reported condom-use for both unknown and negative HIV status were compared with Yc-DNA results.

**Table 2:** Comparison of Yc-DNA results with Self-Reported Condom-use with All Partners in the Past Month

Condom Use W/ All Partners in the Past Month	Yc-DNA Result		
	Negative n(row%)	Positive n(row%)	Total n(col%)
Did Use Condom	25(65.8)	13(34.2)	38(9.22)
Did Not Use Condom Every Encounter	193(51.6)	181(48.4)	374(90.78
Total n(row%)	218(52.91)	194(47.1)	412(100)

When comparing self-reported condom-use with all partners in the past month, 6.07% of all participants who reported consistent condom use had a negative Yc-DNA result. Furthermore 34.2% of participants who reported consistent condom-use with all partners in the past month had a positive Yc-DNA result. There were 193 participants who reported they did not use a condom every encounter but had a negative Yc-DNA result. Of the participants who reported inconsistent condom-use 48.4% had a positive Yc-DNA result.

**Table 3:** Comparison of Yc-DNA results with Self-Reported Condom-use with Partners whose HIV status was Unknown in the Past Month

Condom Use W/ Partners Whose HIV Status Were Unknown In The Past Month	Yc-DNA Result		
	Negative n(row%)	Positive n(row%)	Total n(col%)
Did Use Condom	108(52.9)	96(47.1)	204(56.98)
Did Not Use Condom Every Encounter	79(51.3)	75(48.7)	154(43.02)
Total (row%)	187(52.2)	171(47.8)	358

The comparison between self-reported condom-use in the past month, with partners whose HIV status was unknown, showed that although 204 participants reported using a condom for every sexual encounter, 96 (47.1%) had a positive Yc-DNA result. Although 154 participants indicated they did not use a condom for every sexual encounter, 79 (51.30%) participants had a negative Yc-DNA result.

**Table 4:** Comparison of Yc-DNA result with Self-Reported Condom-use with Partners Who were HIV Negative In The Past Month

Condom Use W/ HIV Negative Partners in the Past Month	Yc-DNA Result		
	Negative n(row%)	Positive n(row%)	Total n(col%)
Did Use Condom	28(62.2)	17(37.8)	45(16.4)
Did Not Use Condom Every Encounter	119(51.7)	111(48.3)	230(83.6)
Total n(row%)	147(53.5)	128(46.5)	275

When comparing self-reported condom use with partners who were HIV negative in the past month with Yc-DNA results, 111 (48.3%) participants reported condomless sex and received a positive Yc-DNA result. Of the 230 (83.6%) participants who reported engaging in condomless sex in the past month, 119 (51.7%) received a negative Yc-DNA result. There were 17 (37.8%) participants who reported using a condom for every sexual encounter yet received a positive Yc-DNA result. The majority (62.2%) of participants who reported using a condom for every sexual encounter with partners who were HIV negative had a negative Yc-DNA result.

**Table 5:** Comparison of Self-Reported Condom-Use with Partners Who were Negative in the Past Month and Self-Reported Condom-Use with Partners whose HIV Status was Unknown in the Past Month

Condom Use with Partner whose HIV Status is Unknown in Past Month	Condom use with Partner who is HIV Negative in past Month			
	Did use condom n(row%)		Did not use condom n(row%)	Total n(col%)
Did use condom	56(23.2)		185(76.8)	241(58.9)
Did not use condom every encounter	12(7.1)		156(92.9)	168(41.1)
Total (row%)	68(16.6)		341(83.4)	409
McNemar χ²	151.92	**McNemar χ² p-value**		P < 0.001

The McNemar Chi-squared test comparing self-reported condom-use with partners who were HIV negative and partners whose HIV status was unknown resulted in strong evidence (p < 0.001) for a difference between the two measures. The difference in distribution of self-reported measures can be seen in Table 5. Only 13.7% (n = 56) reported using a condom for every sexual encounter in the past month with partners who were both HIV negative and whose HIV status was unknown. The majority (n = 185, 45.2%) of participants reported not using a condom with partners who did not have HIV, while they did use a condom with partners whose HIV status was unknown. A significant number (n = 156, 38.1%) did not use a condom for every sexual encounter, regardless of HIV status.

Further paired significance testing was conducted to compare self-reported condom use and Yc-DNA results. These results, as well as sensitivity, specificity, and positive predictive value for both condom-use with partners who were HIV negative and partners whose HIV status was unknown are shown in Table 6.

**Table 6:** Results For Analysis Comparing Yc-DNA With Self-Reported Condom-Use With Both Partner Who Is HIV Negative And Partner Whose HIV Status Is Unknown In The Past Month

	HIV Negative	HIV Status Unknown
McNemar-Bowker test P-value	P < 0.001	P < 0.001
Cohen’s Kappa	-0.0153	-0.0067
Agreement	20.70%	32.00%
Sensitivity (95%CI)	51.7 (95% CI 44.0, 59.3)	49.5 (95% CI 43.9, 55.2)
Specificity (95%CI)	37.8 (95% CI 30.4, 45.2)	47.1 (95% CI 41.5, 52.7)
Positive Predictive Value(95%CI)	68.9 (95% CI 61.8, 76.0)	31.7 (95% CI 26.4, 36.9)
Sample Size	275	358

The McNemar-Bowker test indicates strong evidence for a difference between the results of Yc-DNA and self-reported condom-use with both HIV negative partners (p < 0.001) and with partners whose HIV status was unknown (p < 0.001). The negative values of Cohen’s Kappa indicates that there is disagreement between Yc-DNA and self-reported condom-use with both partners who were HIV negative (-0.0153) and partners whose HIV status was unknown (-0.0067). This is reiterated in low percentage agreement for self-reported condom-use with partners who were HIV negative (20.7%), and partners whose status was unknown (32.0%).

### Log Binomial Model for Factors Associated with Yc-DNA and Self-Report

#### Factors Associated with Yc-DNA Results

Initial bivariate log binomial testing of the association of Yc-DNA with each characteristic indicated no evidence for association for all variables. The multivariate log binomial model for Yc-DNA results indicated evidence for an association with age, after adjusting for age and HIV status of the most recent sexual partner. Those aged 22–24 were more likely to have a positive Yc-DNA test compared to those aged 15–18 (RR:1.36 95%CI 1.04,1.76 p = 0.023). There was also evidence for an association of Yc-DNA results with those of “Other Religion”. They had an increased risk of 1.64 (95%1.06, 2.50 p = 0.024). No evidence for other statistically significant associations were found.

**Table 7:** Descriptive and Multivariate Log Binomial Model of Factors Associated With Positive Yc-DNA Results among Young Women Aged 15–24 in Kampala, Uganda

Variable	Relative Risk (95%CI)	P-value	Adjusted* RR (95%CI)	P-value
Age of Participant				
15–18				
19–21	1.07(0.82,1.40)	0.599	1.16(0.88,1.53)	0.308
22–24	1.20(0.93,1.56)	0.153	1.36(1.04,1.76)	0.023**
Level of Education				
No Education				
Primary Level	0.94(0.75,1.19)	0.630	0.93(0.73,1.17)	0.547
Secondary or Tertiary	0.90(0.68,1.19)	0.462	0.88(0.66,1.18)	0.383
Religion				
Born Again				
Roman Catholic	1.06(0.77,1.48)	0.714	1.08(0.77,1.50)	0.655
Moslem	0.95(0.67,1.36)	0.764	1.00(0.70,1.43)	0.993
Protestant	1.24(0.87,1.75)	0.236	1.25(0.88,1.79)	0.220
Other Religion	1.51(0.88,2.59)	0.138	1.64(1.06,2.50)	0.024**
No Religion	1.13(0.41,3.13)	0.815	0.91(0.18,4.56)	0.907
Most Recent Sexual Partner				
Casual				
Steady	1.17(0.61,1.51)	0.231	1.17(0.91,1.51)	0.231
Spouse	1.15(0.89,1.47)	0.284	1.09(0.83,1.42)	0.540
Most Recent Sexual Partners HIV Status				
HIV Positive				
HIV Negative	3.62(0.59,22.34)	0.166	3.77(0.60,23.84)	0.159
Don’t Know	3.06(0.50,18.91)	0.229	3.07(0.49,19.46)	0.233
Condomless Sex with Partner who is HIV Negative in Past Month				
Never				
Between 1–5	1.54(0.76,3.12)	0.227	1.19(0.76,1.78)	0.400
Between 5–10	1.55(0.72,3.36)	0.266	1.22(0.79,1.87)	0.363
More than 10	1.50(0.62,3.50)	0.344	1.24(0.78,2.00)	0.362
Don’t Know	1.34(0.66,2.73)	0.421	1.20(0.78,1.86)	0.400
Condomless Sex with Partner with Unknown HIV Status in Past Month				
Never				
Between 1–5	1.07(0.84,1.37)	0.568	1.10(0.87,1.40)	0.410
Between 5–10	0.96(0.63,1.45)	0.846	1.09(0.73,1.63	0.657
More than 10	0.97(0.60,1.56)	0.887	1.05(0.65,1.68)	0.847
Don’t Know	0.80(0.52,1.22)	0.296	0.87(0.57,1.32)	0.512

*Adjusted for Age and Most Recent Sexual Partners HIV Status

### Self-Reported Condom-Use with Partners Whose HIV Status Was Unknown

**Table 8:** Multivariate Log Binomial Models For Factors associated Condomless Sex With Partners Whose HIV Status are Unknown

Variable	Did use condom (row%)	Relative Risk (95%CI)	P-value	Adjusted* RR (95%CI)	P-value
Age of Participant					
15–18	90 (52.6)				
19–21	117(54.4)	0.96(0.78,1.19)	0.726	0.98(0.79,1.22)	0.854
22–24	96 (58.9)	0.87(0.68,1.11)	0.251	0.90(0.70,1.15)	0.396
Level of Education					
No Education	105(49.3)				
Primary Level	125(57.1)	0.85(0.69,1.04)	0.106	0.84(0.68,1.02)	0.082
Secondary or Tertiary	73 (62.4)	0.74(0.57,0.97)	0.029**	0.76(0.58,0.99)	0.043**
Religion					
Born Again	37 (52.1)				
Roman Catholic	112(53.1)	0.98(0.74,1.30)	0.887	0.98(0.74,1.30)	0.900
Moslem	93(57.8)	0.88(0.65,1.19)	0.416	0.87(0.64,1.17)	0.353
Protestant	54(57.5)	0.89(0.63,1.25)	0.493	0.90(0.64,1.13)	0.558
Other Religion	7(70.0)	0.63(0.24,1.66)	0.348	0.62(0.23,1.65)	0.337
No Religion	0 (0.0)	1	-	1	-

*adjusted for age, religion and level of education

** strong evidence found

Initial descriptive log binomial models for self-reported condom-use with partners whose HIV status is unknown highlighted some evidence (p = 0.029) for an association with the level of education. The relative risk of reporting condomless sex was 0.76 (95%CI 0.58, 0.99) for participants who completed secondary or tertiary education compared to those with no education, after adjusting for confounding. No other evidence for an association with demographic factors was found.

Subsequently, a multivariate log binomial model controlling for age, religion and level of educational attainment was conducted to identify factors associated with self-reported condomless sex with partners with unknown HIV status. The relationship between self-reported condom-use and level of education was retained after adjusting for confounding (RR 0.76 95%0.58, 0.99 p = 0.043). No other statistically significant relationships were detected. No evidence for statistical interaction or confounding was found.

### Self-Reported Condom-Use with Partners Who Are HIV Negative

**Table 9:** Binomial Regression Models for Factors Associated With Condomless Sex With Partners Who are HIV Negative

Variable	Did Use Condom (row%)	Relative Risk (95%CI)	P-value	Adjusted* RR (95%CI)	P-value
Age					
15–18	20(14.7)				
19–21	27(17.0)	0.97(0.88,1.07)	0.593	0.92(0.86,0.99)	0.017**
22–24	22(17.1)	0.97(0.88,1.08)	0.602	0.99(0.99,0.99)	0.009**
Level of Education					
No Education	30(17.5)				
Primary Level	25(14.8)	1.03(0.94,1.13)	0.491	1.02(0.94,1.10)	0.660
Secondary or Tertiary	14(16.7)	1.01(0.90,1.14)	0.860	1.08(1.01,1.15)	0.028**
Religion†					
Born Again	2 (3.4)				
Roman Catholic	29(18.0)	0.85(0.78,0.93)	0.000**	0.81(0.75,0.87)	0.000**
Moslem	19(16.4)	0.87(0.79,0.95)	0.003**	0.82(0.75,0.89)	0.000**
Protestant	15(18.8)	0.84(0.75,0.94)	0.003**	0.81(0.73,0.90)	0.000**
Other Religion	3(50.0)	0.52(0.23,1.15)	0.107	0.49(0.22,1.10)	0.084
No Religion	1 (100.0)	1	-	1	-

*Adjusted for religion  ** Statistically significant

†Results for religion was adjusted for age

Bivariate log binomial models for self-reported condom-use with partners who were HIV negative found no association with age or level of education. There was strong evidence found for an association of self-reported condom-use and being Roman Catholic (RR0.85 95%0.78, 0.93 p < 0.000), Moslem (RR 0.87, 95%Ci 0.79, 0.95 p = 0.003) and Protestant (RR 0.84 95%CI 0.75, 0.94 p = 0.03) participants when compared to Born-Again (Pentecostal) participants. Subsequent multivariate log binomial model adjusting for age and religion resulted in failed convergence, indicating that confounding factors contributed to the linear relationships seen in the crude model. When adjusted for confounding by religion, there was evidence for an association between those aged 19–21 (p = 0.014) and 22–24 (p = 0.009) with condomless sex with partners who are HIV negative. Participants aged 19–21 had a reduced relative risk of reporting condomless sex (0.92 95%CI 0.85,0.99) compared to 15–18 year olds. Participants aged 22–24 were less likely to report condomless sex with partners who were HIV negative (RR0.99 95%CI 0.99, 0.99). There was no evidence for statistical interaction or confounding found. Furthermore, evidence was found for an association with an association with secondary or tertiary education level (p = 0.028) after adjusting for religion. Participants who attained secondary or tertiary level education had an 8% increased risk of reporting condomless sex (95%CI 1.01,1.15). The evidence for association of Roman Catholic, Moslem and Protestant participants with condomless sex with HIV negative participants was retained after adjusting for confounding.

### Missing data

Investigation of missingness highlighted complete cases for all variables were observed in 43.2% of participants and these participants would therefore be included if using traditional complete case restriction methodology. Yc-DNA was found to be missing in 22.7% of participants. Yc-DNA was missing from participants who reported menstruation at the time of swab collection, or results from swab were invalid due to insufficient DNA found. Missing data for self-reported condomless sex with partners who were HIV negative was 6.5%. The degree of missing data regarding self-reported condom-use with partners who were HIV negative was not explained. Sensitivity analysis was conducted by imputing data of participants with missing variables. These results were similar to the results from complete case restriction.

## Discussion

This study reports on the agreement between the biomarker Yc-DNA and self-reported condomless sex among YWSS aged 15–24 in Kampala, Uganda. The findings indicate strong evidence for a difference between self-reported condom-use and Yc-DNA biomarker results. As validity of Yc-DNA has been established through previous research [3], our results suggest that there was significant discordance in the reporting or detection of condomless sex in this study. Although overall self-reported condom-use was low, the majority of participants had a negative Yc-DNA test.

The results of this study indicated a significant difference between self-reported condom-use and Yc-DNA, potentially reflecting the influence of social-desirability bias. Our results showed a significant proportion of participants who reported they did use a condom with partners whose HIV status was unknown had a positive Yc-DNA result. This discordance of results could be reflective of social desirability bias and has vital clinical and research implications. Furthermore, this result is compounded by the degradation of Yc-DNA over 14 days highlighting a difference between reported and Yc-DNA results. It is this group of participants that may be at highest risk, and it is therefore vital for researchers and clinicians to reduce the impact of social desirability bias on reporting to improve clinical outcomes and the support provided to these girls and women. The decision to engage in a high-risk activity is not solely made in the immediate interaction, but rather reflects several factors such as stigma, financial instability and gendered power imbalances, which interact to facilitate or prevent harm. Therefore, it is important that interventions targeting condom-use reflect and address these broader determinants to enable consistent condom-use.

Self-reported condomless sex with partners who were HIV negative was greater than with partners whose HIV status was not known. This suggests participants knew they should be using condoms with partners whose HIV status was unknown. This form of information bias is the consequence of participants providing answers which are perceived as socially acceptable [17, 23]. This is a complicated bias to conceptualise as participants may be concerned with how their image is observed by the researcher, as well as their perception of self and their morality, resulting in a potential unconscious effect on their answers. Previous studies (VOICE-D and FEM-PrEP) compared biomarker results and self-report also show a lack of congruency [4, 24]. Although ACASI has been shown to reduce the influence of social desirability bias when compared to face-to-face interviews, stigma and social desirability appears to continue to influence self-report in this setting [25]. This form of information bias is the consequence of participants providing answers which are perceived as socially acceptable [26].

Self-reported sexual behaviour may also be influenced by purely methodological constraints, such as recall bias [27]. This is particularly evident when considering sexual behaviour over longer time periods, with research indicating shorter recall periods improving reliability of self-report [18]. Furthermore, meta-analysis indicates that the ability to recall information varies depending on the type of behaviour measured [28]. The difference between Yc-DNA results and self-reported condom-use found in this study could be explained by recall bias, as participants were asked to recall the number of times they had condomless sex over the last month. This is further complicated by the degradation of Yc-DNA with time, causing validity to be limited to 14 days after condomless sex. However research conducted among FSW in Benin indicated that recall did not impact self-reported condom-use and concluded that social desirability bias played a significant role in the reporting of condomless sex [9]. It may also be of note to recognise the impact of the number of sexual partners on the ability to recall condomless sex, limiting the reliability of self-reported results.

The VOICE-D trial, investigating discordance of adherence to oral and vaginal Tenofovir through in-depth interviews, highlighted the role of fear in self-reported product use. The researchers observed that those taking part feared being removed from the trial, thereby losing access to free healthcare provided, as well as fear of repercussion and staff disapproval [4]. The FEM-PrEP study also highlighted fear of being terminated from the study as a factor motivating over-reporting adherence [24]. This research demonstrates the complex relationship developed between researchers and participants, particularly when conducted in already stigmatised and marginalised communities. It could be that fear of removal from the clinic could have influenced self-reported condom-use in this study.

Analysis of Yc-DNA results, and thereby condomless sex, indicated an association with age and HIV status of the most recent sexual partner. Similar results were found in a study among older FSW in Vietnam [29], who perceived themselves to be at lower risk due to their older clientele. The authors suggested that the higher odds of condomless sex among older FSW in Vietnam may be due to their perceived lack of “desirability”, constraining their ability to negotiate condom-use [29].

When considering factors associated with self-reported condom-use it may be seen that socio-cultural factors can influence self-reported condom-use. In our study, having a higher level of education was found to be associated with higher self-reported condom-use with partners whose HIV status was unknown. However, an association between Yc-DNA results and education was not found in the log binomial model for Yc-DNA, suggesting that participants who had higher educational attainment answered in the socially acceptable way. Although previous research has highlighted reduced condom-use among sex workers with lower levels of education [30], no association with discordance of biomarker and self-report with education level has been found [31]. Level of education did not impact condom-use, as demonstrated by Yc-DNA results, but did alter self-reported data, highlighting the role of social desirability bias.

We found that self-reported condom-use with partners who were HIV negative was associated with religion after adjusting for potential confounding by age. A study conducted in Ghana indicated that condom-use was linked with external societal factors such as socio-economic status, ethnicity, and place of residence rather than religiosity [32]. Conversely, research conducted among groups at high risk of HIV in Uganda [33] and Nigeria [34] has indicated that religion was associated with unsafe sexual behaviours, and previous research indicated that Roman Catholic, Moslem and Protestant participants were more likely to engage in condom-use when compared to those who identify as Born Again.

It is important to acknowledge that although the validity of Yc-DNA has been shown in previous clinical trials mostly conducted in the global north among women in monogamous relationships, the validity may be constrained in a more pragmatic setting such as this study. Given the very time-specific validity of the Yc-DNA test, it limits the applicability of the test to a narrow time window of measurement. In this study examining concordance between Yc-DNA and self-reported data, the Yc-DNA results did not reflect self-reported consistent condom-use, as negative Yc-DNA results were greater than the number of participants reporting consistent condom-use. Yc-DNA results are shown to only reflect condomless sex in the past 14 days, as DNA degrades with time. This could result in chronological bias leading to underestimation of condomless sex, as self-reported measures recorded condomless sex over the past month. Additionally, Yc-DNA may also be found subsequent to other contact with a man such as condom breakage or slippage, digital stimulation, the removal of a condom without consent or partial condom use. This draws into question the applicability of Yc-DNA biomarkers in a complex setting among women whose sexual encounters are more difficult to report than those of the heterosexual monogamous women included in previous studies. The self-collected vaginal swabs are dependent on intrasubject and sampling variability. The PCR Test detected both DYs14, (a Testis-specific Y-encoded protein 1) and GAPDH gene. GAPDH gene was positive for all positive and negative samples. The GAPDH Gene helped us ascertain the integrity of the sample as it demonstrates that the sample was well collected and had sufficient human nucleic acids on the swab to conduct PCR analysis. This prevented the inclusion of inappropriately collected samples.

There are some limitations to our analysis. First, although this study has the largest sample size compared to previous studies on Yc-DNA, there was a significant number of missing and invalid Yc-DNA results. This could result in a non-differential bias and limits the strength of results. The significant number of invalid results were considered to be the result of lack of DNA detected on the swab, due to sample collection or processing. Missing results was attributed to self-reported menstruation. The comparison between Yc-DNA and self-reported condom-use is limited by the difference in time period, leading to an underestimation of condomless sex. Additionally, self-reported data collected were disaggregated for partners HIV status, which could lead to overlap of results when compared to Yc-DNA results, leading to non-differential misclassification. This may have caused an underestimation of condomless sex. This was addressed by aggregating data in the analysis. Finally, additional behavioural factors such as type of sex and vaginal douching previously hypothesized to impact the validity of Yc-DNA were not included in this analysis, leading to an under-estimation of condomless sex detected by Yc-DNA.

## Conclusion

We compared the vaginal swab Yc-DNA biomarker with self-reported condom-use among young women who sell sex in Kampala, a particularly vulnerable and epidemiologically important population. Our findings provide a novel perspective on sexual behaviour research among populations at high risk of HIV infection from a compassionate and nuanced perspective, acknowledging that individual decision-making is influenced by institutional harm production. This paper highlights the discordance of self-reported condomless sex with Yc-DNA results and explores potential explanations for this difference. Condomless sex was reported more frequently by participants when partners were known to be HIV negative. Self-reported condomless sex was low, and most participants did not have a positive Yc-DNA result. Future research should prioritise reducing the influence of social desirability on sexual behaviour reporting.

## Data Availability

Data supporting the conclusions of this article can be retrieved following the data sharing policy at the MRC/UVRI and LSHTM Uganda Research Unit. The policy can be accessed through the MRC/UVRI and LSHTM Uganda Research Unit Head of Data Management (Ayoub Kakande – ayoub.kakande@mrcuganda.org).
